# Robotic docking time with the Hugo™ RAS system in gynecologic surgery: a procedure independent learning curve using the cumulative summation analysis (CUSUM)

**DOI:** 10.1007/s11701-023-01693-w

**Published:** 2023-08-05

**Authors:** Giovanni Panico, Sara Mastrovito, Giuseppe Campagna, Giorgia Monterossi, Barbara Costantini, Alessandro Gioè, Riccardo Oliva, Chiara Ferraro, Alfredo Ercoli, Francesco Fanfani, Giovanni Scambia

**Affiliations:** 1 Dipartimento di Scienze della Salute della Donna e del Bambino e di Sanità Pubblica, Fondazione Policlinico Universitario A. Gemelli IRCCS, UOC Chirurgia Ginecologica, 00168 Rome, Italy; 2 Dipartimento di Scienze della Salute della Donna e del Bambino e di Sanità Pubblica, Fondazione Policlinico Universitario A. Gemelli IRCCS, UOC Ginecologia Oncologica, 00168 Rome, Italy; 3 IRCAD, Research Institute Against Digestive Cancer, 1, Place de l’Hôpital, 67091 Strasbourg, France; 4 PID Ginecologia Oncologica e Chirurgia Ginecologica Miniinvasiva, Università Degli Studi di Messina, Policlinico G.Martino, 98124 Messina, Italy; 5 Dipartimento di Scienze della Salute della Donna e del Bambino e di Sanità Pubblica, Università Cattolica del Sacro Cuore, Fondazione Policlinico Universitario A. Gemelli IRCCS, UOC Chirurgia Ginecologica, 00168 Rome, Italy

**Keywords:** Robotic surgery, Hugo RAS, Robotic docking learning curve, CUSUM

## Abstract

Robot-assisted surgery has been proven to offer improvements in term of surgical learning curve and feasibility of minimally invasive surgery, but has often been criticized for its longer operative times compared to conventional laparoscopy. Additional times can be split into time required for system set-up, robotic arms docking and calibration of robotic instruments; secondly, surgeon’s learning curve. One of the newest systems recently launched on the market is the Hugo™ RAS (MEDTRONIC Inc, United States). As some of the earliest adopters of the Hugo™ RAS system technology, we present our data on robotic docking learning curve for the first 192 gynecologic robotic cases performed at our institution. Our data indicates that robotic set-up and docking with the new Hugo™ RAS robotic surgical system can be performed time-effectively and that the specific robotic docking learning curve is comparable to preexisting data for other platforms. This preliminary insights into this recently released system may be worthwhile for other centers which may soon adopt this new technology and may need some relevant information on topics such as OR times. Further studies are necessary to assess the different features of the Hugo™ RAS considering other technical and surgical aspects, to fully become familiar with this novel technology.

## Introduction

In recent years, robot-assisted surgery (RAS) has emerged as a promising alternative to laparoscopy in both benign and malignant gynecologic disease surgery. RAS has been proven to offer various benefits to both surgeons and patients, such as improvements in term of surgical learning curve and feasibility of minimally invasive surgery (MIS) [1].

The introduction of the first robotic-assisted surgical system in 1999, the daVinci robot (Intuitive Surgical System), established a significant milestone in the evolution and spread of RAS. Since then, it has continued to advance, with new systems and surgical applications being developed and refined.

As with every new technology, while potential benefits have been described over the years, some downsides must be carefully considered [2]. Higher costs, longer operative times, specialized training requirements, and potential human over-reliance on technology should be taken into account as potential limitations [3, 4].

Over the last decade, new robotic systems have entered the surgical marketplace, in order to overcome some of the downsides of RAS [5, 6]. While the daVinci surgical system remains the most widely used and well-known surgical platform, these alternatives offer unique features, and their differences may result in potential advantages [7].

One of the newest systems recently launched on the market is the Hugo™ RAS (MEDTRONIC Inc, United States), designed to provide greater flexibility and control during minimally invasive surgery [8–11]. Some of its features include a remote open surgical console controlling four independent manipulator arms that differ from the monolithic structure of the daVinci Surgical System [8].

As with any new surgical technology, the benefits and limitations of each robotic system should be carefully considered before drawing any conclusion. In particular, their features need to be compared to pre-existing techniques and systems [12].

As forementioned, RAS has often been criticized for its longer operative times compared to conventional laparoscopic surgery. These longer times can be split into two components: first, the additional time required for system set-up, robotic arms docking and calibration of robotic instruments; secondly, the surgeon’s lack of experience with a new robotic system could well lead to longer operative times [13].

While operative time may be a concern, it is essential to note that it is dependent upon a variety of factors, especially in complex cases. As with any new surgical system, an initial learning curve must be overcome in order to achieve proficiency. Many studies have analyzed robotic surgical learning curves on the Da Vinci platform and have suggested that the longer operative times associated with RAS decrease as surgeons become more familiar with the technology [14]. Additionally, in some procedures, added precision conveyed by the robotic instruments and enlarged vision may actually lead to shorter operative times, especially in complex cases [1, 3].

**Fig. 1:**
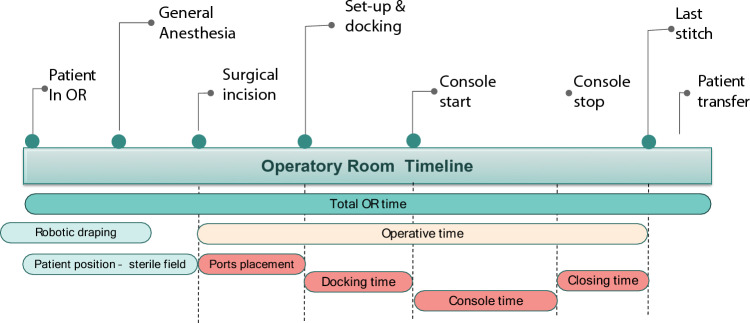
Summary of operating room timeline for a robotic procedure

Although the literature provides an abundance of data about robotic surgical learning curves in gynecologic surgery as well as in other specialties [14], there is a knowledge gap regarding learning curve of phases that precede the actual procedure, such as system set-up and robotic docking (Fig. 1) [15–18]. In particular, the only available data on docking times and their relater learning curves stem from the da Vinci robotic surgical platforms [16, 18–20].

For such reasons, as some of the earliest adopters of the Hugo™ RAS system technology, we report our initial experience on robotic docking learning curve in gynecologic surgery. This preliminary insights into a recently released platform may be valuable to other centers that may soon introduce this new technology and may provide some relevant information on such a major topic such as OR times.

## Materials and methods

We present our data originating from a prospective observational study for the first 192 gynecologic robotic cases performed at our institution from March 2022 to January 2023.

All gynecological procedures performed with the Hugo™ RAS system were included, independently from the procedure type. All cases were performed by 9 different experienced surgeons. Robotic docking was always completed by the first bedside assistant, consisting of 6 well-trained senior residents, who all completed a 3-days hands-on simulation training course dedicated to the use of the Hugo™ RAS. All patients suitable for the planned surgery through a minimally invasive approach were considered eligible for the study. Other general inclusion criteria were the following: age > 18 years, American Society of Anesthesiologists’ (ASA) score up to III, and no absolute contraindications to pneumoperitoneum or Trendelenburg position. Informed consent was obtained from all patients included in this study. All research activities were approved by the Institutional Review Boards. (IRB No. 0012761/22).

Collected data included routine patient demographics information, surgical procedure data and intraoperative data.

During surgery, specific time parameters were measured (Fig. 1).

Preoperative time was defined as the time from the moment when the patient entered the OR to the surgical incision and included time for general anesthesia and patient positioning.

Trocar placement time was defined as the time from the first surgical incision to the positioning of the last trocar. This time included laparoscopic adhesiolysis in cases which required it in order to place all trocars.

Docking time was defined as the time necessary to move the robotic arms into the surgical field, to set the arms into their respective port sites, and to insert the robotic instruments in the abdomen. Operative time was defined as the interval from the start of the procedure to the suturing of the surgical incision. Console time was considered from the moment when the first operator started the procedure at the robotic console, until the end of its usage.

### Robot set-up and docking

All procedures were performed using a standard technique as previously published by our group [8–11]. The robotic arms were covered with adequately shaped sterile drapes by OR nurses before the start of the procedure. After general anesthesia procedures were completed, patients were positioned in a dorsal lithotomy position. All procedures, independently from the surgery type, started with the insertion of a 12-mm optical port placed transumbilically. Once the pneumoperitoneum was established, a 3D-HD 0-degree, 10 mm scope (Karl Storz Endoscopy) was inserted. Two to three additional 8 mm robotic ports were placed under direct vision in the right and left lower abdomen following the “compact” scheme configuration previously published by Gueli Alletti et al. [8]. As for docking, to position each arm, two main settings were required, and could be adjusted depending on patients’ characteristics: (1) the tilt angle—a vertical angle between the arm and the operative field; (2) the docking angle—a clockwise horizontal angle between the patient’s head and the arm’s direction [11]. Small adjustments were made during docking to optimize the angles necessary for each patient.

### Statistical analysis and CUSUM analysis

Data were analyzed using the SPSS (version 29.0; SPSS Inc, Chicago, IL, United States). Means ± SD, medians, ranges, and percentages were used as descriptive statistics. Univariate linear regression was performed with the docking time as a dependent variable, as a function of consecutive cases as independent variable. Statistical significance was considered at *p* value: 0.01. The learning curve of docking time was assessed with cumulative summation analysis (CUSUM). It was first described by E.S. Page in 1954 as a sequential analysis technique to assess productivity and process improvement and could be explained as a running total of the sum of the deviations of individual samples from a prespecified target [21]. Nowadays, it is an established method to represent data from consecutive procedures, transforming raw data into a cumulative sum of differences between single values and the overall mean [22]. An inflexion point in the trend has been described in the literature as the transition from a learning phase to a proficiency phase. The target used for the docking time was the mean average of operative time for every assistant surgeon individually. Using this method, a learning curve of a surgical procedure is graphically represented as a bell-shaped curve, with the first phase representing the procedures which took longer than the overall mean, and the second downward phase showing the procedures which took less time than the mean. Albeit not a perfect method, it graphically shows a good approximation of the number of procedures required to achieve proficiency, which is shown by a continuously declining graph or a graph maintained at 0 [23].

## Results

Data on surgery type and robotic configurations are summarized in Table 1.

**Table 1:** Surgical procedures and robot configurations

Diagnosis, *N* (%)	
Pelvic organ prolapse	81 (42.2)
Uterine fibromatosis	62 (32.3)
BRCA mutations	20 (10.4)
Gynecologic malignancies	16 (8.3)
Endometriosis	8 (4.2)
Total	**192 (100)**
Associated surgical procedures, *N* (%)	
Sacral colpopexy	81 (42.2)
Hysterectomy	170 (88.6)
Total	94 (49)
Subtotal	76 (39.6)
Adnexectomy	156 (81.3)
Salpingectomy	24 (12.5)
Oncological staging	16 (8.3)
Endometriosis eradication	8 (4.2)
Robotic configuration, *N* (%)	
Compact mode	192 (100)
Robotic arms, N (%)	
3	179 (93.2)
4	13 (6.8)

The main diagnosis was pelvic organ prolapse (42.2%), followed by uterine fibromatosis (32.2%), BRCA mutations (10.4%), malignancies (8.3%), and endometriosis (4.2%). In most cases, multiple procedures were performed during surgery. The most common procedures included hysterectomy (88.6%) and adnexectomy (81.3%), shortly followed by sacral colpopexy (42.2%)

In 93.5% of surgeries, a 3-arm configuration was chosen by the surgical team. A “compact” configuration was chosen in all 192 cases [8]

Data on OR times is summarized in Table 2

**Table 2:** Intraoperative timings

Variable	Mean (min) ± sd	(Range)	Mean percentage
Docking time	5.08 ± 1.99	(2–12)	3.5
Console time	111.49 ± 40.16	(20–235)	68
Port placement + closing time	44.83 ± 21.44	(10–137)	28
Operative time	161.4 ± 49.42	(50–305)	100

Mean operative time. (OT) was 161 min (range 50–305). Mean console time was 111 min (range 20–235), and represented, on average, 68% of the OT. Mean docking time was 5.08 min (range 2–12), representing from 2 to 10% of the OT. By subtracting console time and docking time to the overall OT, we were also able to calculate the time required for port placement, exploratory laparoscopy, and the time from when the surgeon stopped using the console up to the last stitch. The mean of this “opening and closing” time was 44 min (range 10–137), and it represented 28% of the overall OT and it included time for laparoscopic adhesiolysis in cases that required it, and time for specimen extraction from the abdomen.

**Fig. 2:**
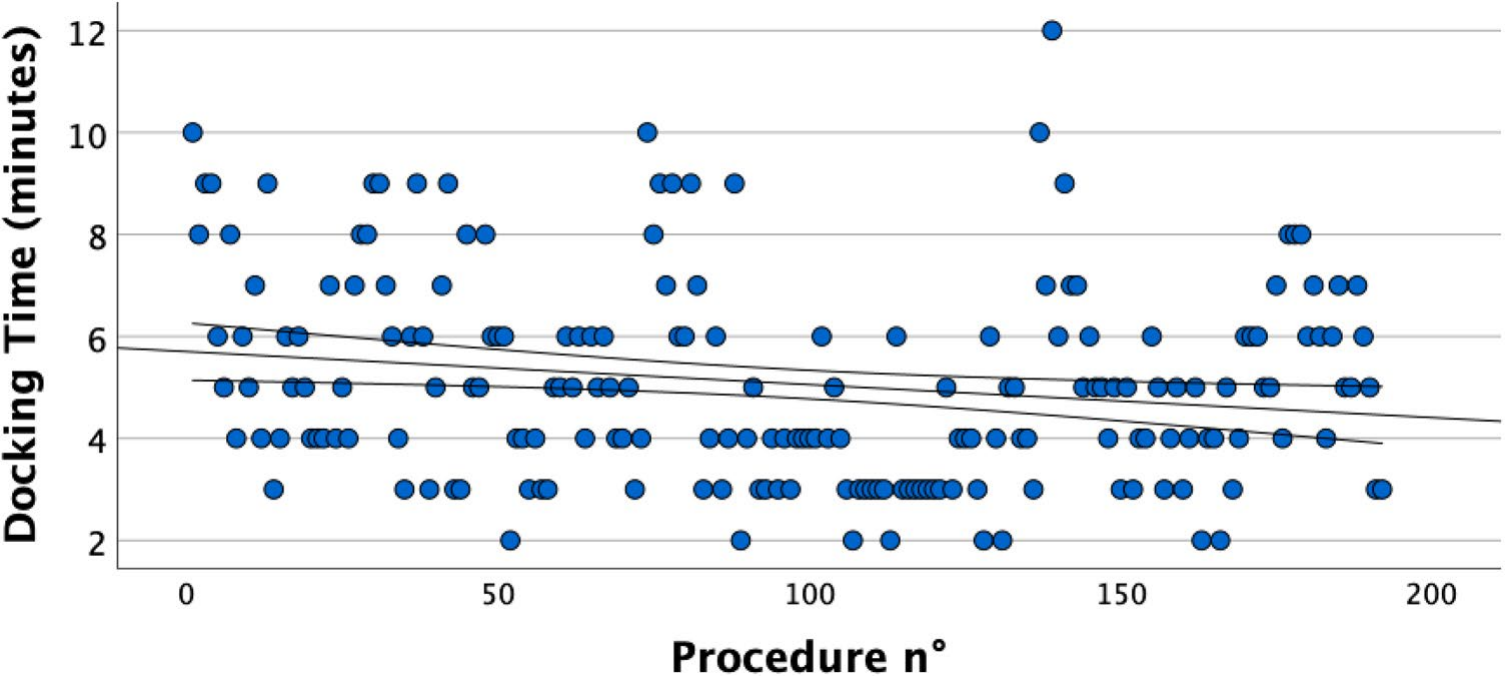
Negative linear correlation between the decrease in docking time and the number of consecutive procedures

The scatterplot in Fig. 2 shows the overall docking time for the first 192 procedures performed at our institution. The line represents the linear regression of the docking time function of the number of performed cases. There was an overall negative linear correlation (Pearson’s r – 0.181, p = 0.006) between the decreasing docking time and the number of consecutive procedures, regardless of the assistant surgeon involved in the procedure.

**Fig. 3:**
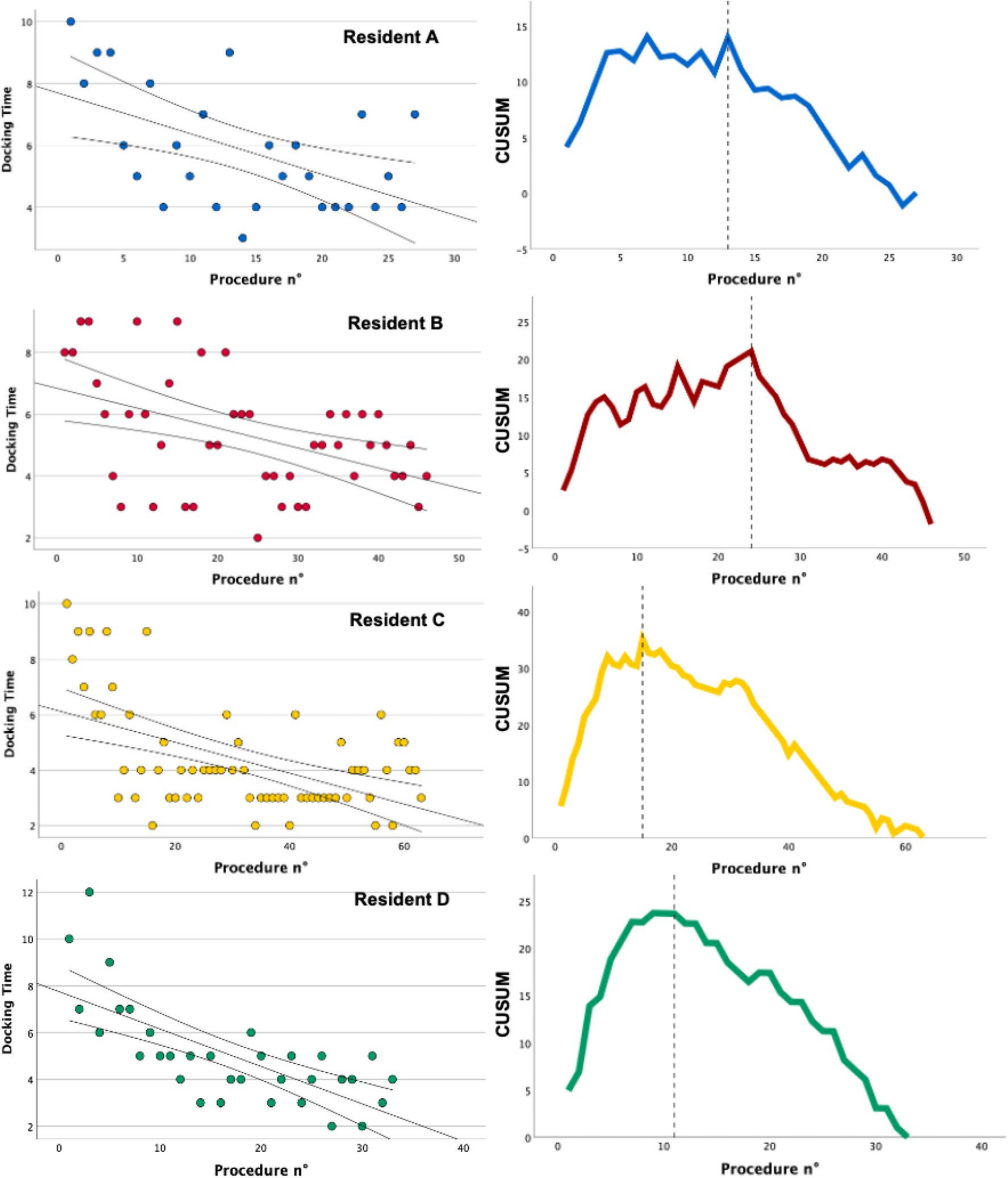
Right column: negative linear correlation between the decreasing docking time and the number of consecutive procedures for each assistant surgeon. Left column: CUSUM curves for each assistant surgeon; dotted line shows the inflection point of the curve, indicating the end of the learning phase

Next, we individually analyzed the learning curves of the 4 assistant surgeons who performed more than 25 procedures (Resident A, B, C, D), to allow the identification and comparison of individual trends amongst them. Mean docking time did not statistically differ between the 4 assistant surgeons (p values > 0.5) and between the 3 and 4 arms configuration (p value > 0.4). Univariate linear regression was performed for each resident with the docking time as a dependent variable and case number as independent variables. There was a significant negative linear correlation between the decreasing docking time and the number of consecutive procedures for each resident (Resident 1: Pearson’s r – 0.535, p = 0.002; Resident 2: Pearson’s r – 0.457, p < 0.001; Resident 3: Pearson’s r – 0.525, p < 0.001; Resident 4: (Pearson’s r – 0.711, p < 0.001) (Fig. 3). The inflection points of the CUSUM curves, indicating the switch from the learning phase to the proficiency phase, is graphically represented at 13 procedure (Fig. 3).

Comparisons between mean times before and after the inflection points of the learning curves were statistically significant (*p* value < 0.01); (Table 3).

**Table 3:** Docking time (minutes) per assistant

Assistant surgeon	Cases (%)	Mean ± sd	Median (range)	Mean (learning)	Mean (proficiency)	P value
Resident A	27 (14.1)	5.85 ± 1.95	5 (3–10)	6.92 ± 2.06	4.86 ± 1.23	**0.009**
Resident B	46 (24)	5.33 ± 1.89	5 (2–9)	6.21 ± 2.04	4.36 ± 1.14	**0.002**
Resident C	63 (32.8)	4.33 ± 1.94	4 (2–10)	6.17 ± 2.47	3.6 ± 1.01	** < 0.001**
Resident D	33 (17.2)	5.03 ± 2.17	5 (2–12)	7.18 ± 2.27	3.95 ± 1.05	** < 0.001**
Resident E	7 (3.6)	5.57 ± 0.97	6 (4–7)	\	\	\
Resident F	16 (8.3)	5.88 ± 1.62	6 (3–8)	\	\	\
Total	**192 (100)**	**5.08 ± 1.99**	**5 (2–12)**	\	\	\

## Discussion

Over the last few decades, the popularity of minimally invasive surgery has increased exponentially. Its primary aim was the reduction of invasiveness and complications related to laparotomic surgery, while maintaining its efficacy and safety.

As forementioned, one of the main arguments against robotic surgery remains prolonged operative time, leading not only to a higher invasiveness for patients, but also to higher costs per procedure. Prolonged overall operating room times due to the use of robotic systems, have been detected by numerous studies over the years [24].

The preparation and execution of robotic docking can be considered as “standard” extra time if compared to laparoscopy, and there is a need to define its magnitude, particularly when adopting a new device that differs from the standard da Vinci monolithic robotic system.

Additionally, in the Hugo™ RAS platform as well as in other “multi-arm” platforms, docking is a fundamental step and it is essential that it is performed correctly to allow maximal instrument freedom of movement, preclude arm collision, and give adequate access to the surgical field through robotic instruments, guaranteeing a smooth and efficient surgery [8, 25].

Previous studies performed using the da Vinci robotic surgical system found docking times ranging from 7 to 22 min, and estimated learning curves comprised between 18 and 60 procedures [16, 20].

To our knowledge, this study is the first to analyze the learning curve of this specific parameter in the novel Hugo™ RAS platform, as well as in any other platform different from the da Vinci’s.

To best graphically show the docking learning curve and the transition from a learning phase to a proficiency phase, we applied the CUSUM chart.

The CUSUM technique has been used in the surgical field to analyze learning curves for surgical procedures for many years. One of its advantages is the ability of detecting small shifts in the system, and to allow continuous analysis in time and rapid graphic evaluation of data. On the other hand, its main downside and critique point is the tendency to over-interpret its results, particularly if times are plotted against the overall mean, which leads to a bell-shaped curve in most of the cases [23]. Although not a perfect method, it graphically showed a good approximation of the number of procedures required to achieve proficiency, as demonstrated by the different shapes of the curves and different inflection points of the different assistant surgeons shown in Fig. 3.

Our overall mean docking time was 5.08 min whereas single assistant surgeons mean docking time ranged from 4.33 to 5.88 min, representing from 2 to 10% of OT.

The outlined data indicate that robotic set-up and docking can be performed time-effectively, even in a platform with single independent arms. There was no significant difference in mean docking time in the 13 cases where the surgeon opted for a 4 arms configuration, but this data may be confounded by the small number of those cases.

Our series found docking time to represent less than 10% of the overall OT, and the learning curve to reach proficiency is likely comprised between 12 and 23 procedures, which does not necessarily affect the overall time spent in the OR.

Although our data suggests shorter times compared to previous studies, unlike those, our “docking time” did not include port placement, as this step is common to conventional laparoscopy and should not be considered as an additional time of robotic surgery[16, 19, 20].

Additionally, OR team efficiency must be taken into account when looking at such data. In fact, all surgeons and assistants included in this study had completed a training program straight before the introduction of the system in clinical practice, and successively participated in a large number of cases in a relatively short period of time. This might not be always true in different scenarios, and slightly longer docking times might be more realistic.

## Conclusions

Our data indicates that robotic set-up and docking with the new Hugo™ RAS robotic surgical system can be performed time-effectively and that the specific robotic docking learning curve is comparable to preexisting data for other platforms. This preliminary insights into this recently released system may be worthwhile for other centers which may soon adopt this new technology, and which may need some relevant information on topics such as OR times. Further studies are necessary to assess the different features of the Hugo™ RAS considering other technical and surgical aspects, to fully become familiar with this novel technology.
